# β3-Adrenergic Signaling Preserves Postnatal Maturation of the Enteric Nervous System and Gut Microbiome During Neonatal Hyperoxia

**DOI:** 10.3390/biom16091284

**Published:** 2026-09-04

**Authors:** Patrizia Nardini, Sara Bertorello, Luca Filippi, Virginia Zizi, Ida Cioffi, Francesco Cei, Simone Baldi, Daniele Bani, Maura Calvani, Camilla Fazi, Amedeo Amedei, Alessandro Pini

**Affiliations:** 1 Department of Experimental and Clinical Medicine, University of Florence, 50134 Florence, Italy; patrizia.nardini@unifi.it (P.N.); sara.bertorello@unifi.it (S.B.); virginia.zizi@unifi.it (V.Z.); ida.cioffi@unifi.it (I.C.); francesco.cei1@unifi.it (F.C.); simone.baldi@unifi.it (S.B.); daniele.bani@unifi.it (D.B.); amedeo.amedei@unifi.it (A.A.); 2 Neonatal Intensive Care Unit, Emergency Department, University Children’s Hospital Meyer IRCCS, 50134 Florence, Italy; luca.filippi@meyer.it; 3 Department of Oncoematology, University Children’s Hospital Meyer IRCCS, 50134 Florence, Italy; maura.calvani@meyer.it; 4 Department of Neurosciences, Psychology, Drug Research and Child Health, University of Florence, 50134 Florence, Italy; camilla.fazi@unifi.it

**Keywords:** β3-adrenergic receptor, hyperoxia, colon, microbiome, enteric nervous system, prematurity

## Abstract

Oxygen availability is a key regulator of organ maturation during the perinatal period. Disruption of physiological oxygen homeostasis contributes to prematurity-associated disorders, yet its effects on the coordinated maturation of the enteric nervous system (ENS) and gut microbiome remain poorly understood. Because β3-adrenergic receptor (β3-AR) signaling has emerged as a mediator of tissue adaptation to oxygen, we investigated whether activation of this pathway modulates hyperoxia-induced alterations in the developing colon. Newborn rats were exposed to normoxia or hyperoxia (85% O_2_) from birth to postnatal day 14 and treated with the β3-AR agonist BRL37344 (1 or 3 mg/kg). Enteric neuronal and glial populations were evaluated by quantitative immunofluorescence, whereas the colonic microbiome (CM) was characterized by 16S rRNA gene sequencing. Hyperoxia reduced neuronal density and altered neurochemical coding within the submucosal plexus, disrupted enteric glial organization in both the colonic submucosal plexus and mucosa, and remodeled the intestinal microbiome without affecting overall community diversity. BRL37344 treatment partially preserved submucosal neurochemical coding, modulated neuron–glia organization within the submucosal plexus, prevented the loss of mucosal enteric glial cells, and reshaped microbial composition. Collectively, these findings demonstrate that neonatal hyperoxia disrupts coordinated postnatal maturation of the ENS and CM and indicate that β3-AR signaling may contribute to postnatal intestinal adaptation to neonatal oxygen imbalance.

## 1. Introduction

Oxygen is a key regulator of organ maturation during the perinatal period [1,2]. The transition from fetal to neonatal life is characterized by a marked increase in oxygen availability, which contributes to the completion of developmental programs initiated during gestation under physiologically hypoxic conditions. Alterations in physiological oxygen levels during this critical developmental window disrupt organ maturation and contribute to neonatal disorders associated with prematurity, including bronchopulmonary dysplasia (BPD), necrotizing enterocolitis (NEC), and retinopathy of prematurity (ROP) [3,4,5,6].

Among the molecular pathways involved in oxygen-dependent developmental adaptation, β3-adrenergic (β3-AR) signaling has recently emerged as a potential mediator linking oxygen availability to tissue maturation [7]. Beyond its established lipolytic actions [8], β3-AR is increasingly recognized as a regulator of cellular proliferation and differentiation in several fetal and neonatal organs, where its expression is influenced by local oxygen [9,10,11,12,13]. Studies in neonatal models of oxygen-induced injury have shown that hyperoxia is associated with a reduction in β3-AR-expressing cells, and pharmacological activation of this receptor attenuates tissue damage and promotes a nearly normal maturation of developing organs, including the lung and intestine [7,10,13,14,15].

The intestine is particularly vulnerable to alterations in oxygen availability during early postnatal life [7,16,17], as its maturation continues extensively after birth, when it must rapidly acquire its numerous functions in an oxygen environment markedly different from that experienced during fetal life. This process requires the coordinated maturation of the epithelial barrier, immune system, enteric nervous system (ENS), and gut microbiome [18,19,20,21,22]. The latter is increasingly recognized as a major driver of postnatal intestinal development, promoting barrier formation, shaping immune development, and contributing to the maturation of the ENS [22,23,24]. In detail, microbiome-derived signals contribute to the postnatal maturation of neuronal [25] and enteric glial populations within ganglionic plexuses and lamina propria [26], regulating the development of enteric neuronal circuits. Because the microbiome is strongly influenced by oxygen availability [27], alterations in oxygen levels during early life may impair the establishment of the intestinal microbial ecosystem. Accordingly, marked dysbiosis has been consistently reported in neonatal models of hyperoxia-induced intestinal injury [27], potentially interfering with normal intestinal maturation [5,17,28].

Despite growing evidence supporting a protective role of β3-AR signaling in hyperoxia-induced intestinal injury, its potential impact on the gut microbiome remains unknown. This question is particularly relevant given the central role of microbial colonization in postnatal intestinal maturation and ENS development. Furthermore, whether β3-AR activation influences the maturation of neuronal and enteric glial populations within the submucosal plexus under hyperoxic conditions remains to be investigated. Therefore, we examined the effects of β3-AR agonism on colonic microbiome (CM) composition and on the neuronal and enteric glial components of the submucosal plexus and lamina propria in neonatal rats exposed to hyperoxia.

## 2. Materials and Methods

### 2.1. Animal Model and Pharmacological Treatment

Timed-pregnant Sprague–Dawley rats (Crl:CD(SD)) were housed individually under standardized environmental conditions, with ambient temperature maintained at 25 ± 2 °C. Animals were kept on a 12 h light/12 h dark cycle and had ad libitum access to food and water. After birth, litters were pooled and randomly allocated to either normoxic (21% O_2_) or hyperoxic conditions. Hyperoxia was maintained in a dedicated chamber (BioSpherix, Parish, NY, USA) supplied with 85% oxygen, and oxygen concentration was continuously monitored using a ProOx P110 sensor (BioSpherix).

The neonatal pups of the treatment groups received subcutaneous injections of the commonly used β3-AR agonist BRL37344 (Merck, Darmstadt, Germany) every 12 h from postnatal day 0 (P0) to postnatal day 14 (P14). To minimize maternal oxygen toxicity, nursing dams were alternated between normoxic and hyperoxic environments every 24 h.

Pups included in the study were obtained from four litters. At P0, pups from all litters were pooled, randomly assigned to experimental groups, and subsequently redistributed among nursing dams to ensure representation of different litters across experimental conditions. Sex was recorded at the end of the experimental period, as reliable determination was not feasible at P0, but it was not included as an independent variable due to the limited number of animals of each sex within individual groups. Neonates were assigned to five experimental groups: (1) normoxia control group, consisting of untreated pups maintained under room air conditions (21% O_2_); (2) normoxia + BRL37344 (3 mg/kg), comprising pups reared in normoxia and treated with BRL37344 at 3 mg/kg; (3) hyperoxia control group, including untreated pups exposed to 85% O_2_; (4) hyperoxia + BRL37344 (1 mg/kg), including pups maintained under hyperoxic conditions and administered BRL37344 at 1 mg/kg; (5) hyperoxia + BRL37344 (3 mg/kg), consisting of pups exposed to hyperoxia and treated with BRL37344 at 3 mg/kg. The BRL37344 doses were chosen based on their previously demonstrated pharmacologic effects in a similar animal model [7,13,14,15]. At the end of the treatment period, the animals were euthanized by i.p. sodium pentobarbital (800 mg/kg). Segments of the proximal colon were excised approximately 2 cm distal to the cecum, rinsed in 0.1 M phosphate-buffered saline (PBS; pH 7.4), fixed overnight at 4 °C in 4% paraformaldehyde (PFA), dehydrated through graded ethanol, cleared in Bio-Clear (Bio-Optica, Milan, Italy), embedded in paraffin wax, and sectioned.

### 2.2. Immunofluorescent Analyses

Immunofluorescence analyses were performed on transverse sections of the proximal colonic samples. Following deparaffinization and rehydration, antigen retrieval was carried out by incubating sections in Tris-EDTA buffer (10 mM Tris, 1 mM EDTA, pH 9.0) at 90–92 °C for 10 min. To reduce nonspecific immunoreactivity, sections were incubated with 3% bovine serum albumin (BSA)(Merck) prior to antibody application.

Samples were incubated overnight at 4 °C with the primary antibodies listed in Table 1. For double-labeling experiments, sections were exposed overnight to a mixture of appropriately diluted primary antibodies prepared in PBS containing 1% BSA, followed by a 2 h incubation at room temperature with the appropriate fluorophore-conjugated secondary antibodies. Slides were mounted using an aqueous antifade mounting medium containing DAPI (Fluoroshield™, Thermo Fisher Scientific, Waltham, MA, USA). Negative controls were obtained by omitting the primary antibodies.

The immunostained sections were examined using a Leica Stellaris 5 confocal laser-scanning microscope (**Leica Microsystems, Wetzlar, Germany**) equipped with 20× and 63× Plan-Apochromat objectives. The total number of HuC/D-, neuronal nitric oxide synthase (nNOS)-, choline acetyltransferase (ChAT)-, SRY-box transcription factor 10 (SOX10)-, and S100β-positive cells within submucosal ganglia and within lamina propria across the entire tissue section was quantified independently by two blinded investigators (P.N. and V.Z.). Data are expressed as the percentage of nNOS-positive or ChAT-positive neurons relative to the total HuC/D-positive neuronal population and are reported as mean ± standard deviation (SD) from at least three sections per animal.

### 2.3. Microbiome Characterization

Genomic DNA was extracted from Formalin-Fixed Paraffin-Embedded (FFPE) colon tissue samples using the QIAamp DNA FFPE Advanced Kit (Qiagen, Hilden, Germany). A blank paraffin block processed in parallel was included as a negative control. The quality and quantity of the extracted DNA were assessed using a NanoDrop ND-1000 (Thermo Fisher Scientific) and a Qubit Fluorometer (Thermo Fisher Scientific), and then samples were stored at −20 °C until further analysis. Extracted DNA samples were sent to IGA Technology Services (I-33100 Udine, Italy), where amplicons of the V3-V4 variable region of the bacterial 16S rRNA gene were sequenced in paired-end mode (2 × 250 pb) on the Illumina Novaseq platform (**Illumina, Inc., San Diego, CA, USA**).

The resulting raw FASTQ files were processed using the MICCA (v.1.7.2) (MICrobial Community Analysis) software pipeline, and demultiplexed sequence reads were subsequently analysed with QIIME2 (v.2021.4). Cutadapt was used to remove sequences lacking primers, while DADA2 was applied to trim low-quality nucleotides from the forward and reverse reads (--p-trunc-len-f 223 --p-trunc-len-r 220), perform paired-end filtering and merging, and remove chimeric sequences. Potential host-derived sequences were identified by aligning amplicon sequence variants (ASVs) to the mRatBN7.2 rat reference genome using Bowtie2 (v.2.4.4) and were subsequently removed. The remaining reads were imported into QIIME2, where taxonomic assignment was performed using a Scikit-learn multinomial Bayes classifier re-trained on the Silva database (release 138) for the V3-V4 hypervariable region.

The microbial profile of the negative control was compared with those of the biological samples to identify potential paraffin- or processing-associated contaminants. The genera *Methanobrevibacter* and *Kineosporia*, which were detected exclusively in the negative control, were considered potential contaminants and removed from the dataset. As an additional conservative filtering step, genera with an average relative abundance below 0.005% were excluded. Host-derived and Chloroplast sequences were also removed before downstream analyses.

### 2.4. Statistical Analysis

Morphological data are presented as mean ± SD and were obtained from at least five animals per experimental group. The sample size reported for each experiment represents the number of individual animals contributing to that analysis, with each pup considered the experimental unit. Sample size was estimated based on the magnitude and variability of the neurochemical alterations previously observed in the ileal submucosal plexus using the same neonatal hyperoxia model [14], assuming a statistical power of 80% and a two-sided α level of 0.05.

Because the experimental design included oxygen exposure and pharmacological treatment as biologically distinct factors, factorial analyses were performed where appropriate. For the complete 2 × 2 subset comprising normoxic and hyperoxic animals treated with BRL37344 at 3 mg/kg or untreated, data were analyzed using an ordinary two-way ANOVA, with oxygen condition and treatment as independent factors, including the oxygen × treatment interaction in the model. Šídák’s multiple-comparison test was used for post hoc comparisons. Normality of model residuals was assessed using the Shapiro–Wilk test, whereas homogeneity of variance was evaluated by residual diagnostics and Spearman’s rank correlation between predicted values and absolute residuals.

Because a normoxic group treated with BRL37344 at 1 mg/kg was not included in the experimental design, dose effects under hyperoxic conditions were evaluated separately by one-way ANOVA comparing untreated hyperoxic animals with animals treated with BRL37344 at 1 or 3 mg/kg, followed by Dunnett’s multiple-comparison test using the untreated hyperoxic group as the reference. Normality of residuals and homogeneity of variance were assessed using the Shapiro–Wilk and Brown–Forsythe tests, respectively. When the assumption of normality was not satisfied, the Kruskal–Wallis test followed by Dunn’s multiple-comparison test was used.

When the assumption of homogeneity of variance was not satisfied, Brown–Forsythe and Welch ANOVA tests followed by Dunnett’s T3 multiple-comparison test, which do not assume equal variances, were performed. Statistical analyses of morphological data were performed using GraphPad Prism version 9.0 (GraphPad Software, San Diego, CA, USA), with *p* < 0.05 considered statistically significant.

Microbiome statistical analyses were performed in R (v4.2.2) using phyloseq (v1.42.0), vegan (v2.6-4), DESeq2 (v1.38.3), ggplot2 (v3.3.6), and ggpubr (v0.6.0). Rarefaction curves were generated using the rarecurve function in vegan to assess sequencing depth adequacy.

Alpha diversity was assessed using observed ASV richness and the Shannon diversity index. Pielou’s evenness was calculated as E = S/log(R), where S is the Shannon index and R is observed richness. Group differences were evaluated using the Kruskal–Wallis test. Beta diversity was assessed using Hellinger distance on Hellinger-transformed genus-level abundance data and visualized by principal coordinate analysis (PCoA). Statistical differences in community composition were tested using permutational multivariate analysis of variance (PERMANOVA) with 9999 permutations.

Differential abundance analysis was performed using DESeq2 on raw count data. *p* values were adjusted using the Benjamini–Hochberg method, with adjusted *p* < 0.05 considered significant. Features with a grand mean count < 100 were excluded a priori.

## 3. Results

### 3.1. Hyperoxia Alters Neuronal Density and Neurochemical Coding in the Submucosal Plexus

**Figure 1:**
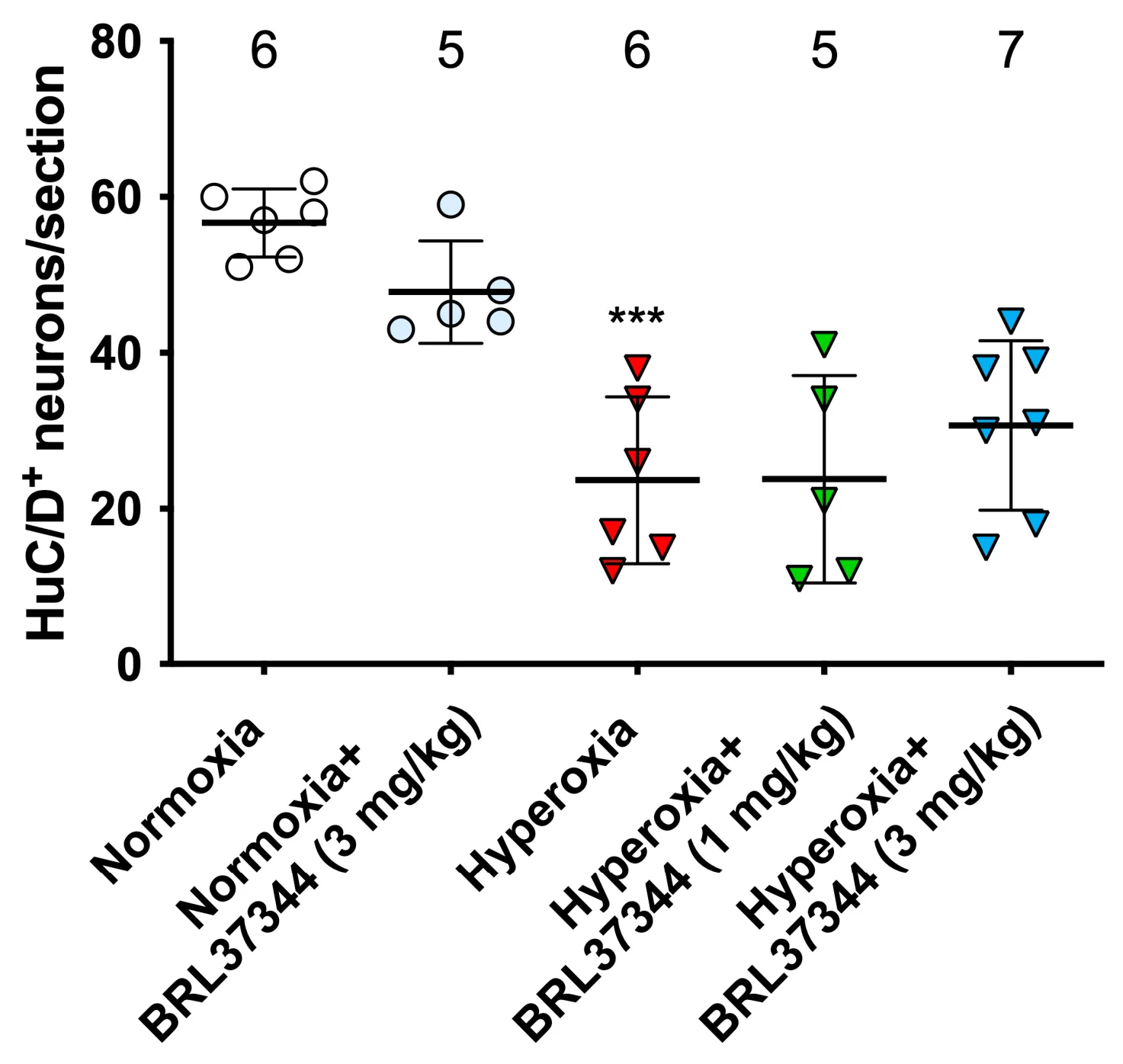
Hyperoxia reduces neuronal density within the colonic submucosal plexus. The pan-neuronal marker HuC/D was used to quantify the total number of enteric neurons per section within the submucosal plexus. Neonatal hyperoxia significantly decreased neuronal density compared with normoxic controls, whereas neither dose of BRL37344 significantly reversed this effect under hyperoxic conditions. Values are expressed as mean ± SD. Statistical analyses were performed using Brown–Forsythe/Welch or ordinary one-way ANOVA, followed by Dunnett’s T3 or Dunnett’s multiple-comparison tests, respectively, as appropriate. *** *p* < 0.001 vs. normoxia.

To assess the effects of hyperoxia on the neuronal compartment of the submucosal plexus, the total neuronal population was quantified using HuC/D immunoreactivity, whereas nitrergic and cholinergic neuronal subpopulations were identified by nNOS and ChAT immunofluorescence, respectively. Hyperoxia exposure significantly reduced the total number of neurons per section compared with normoxic controls. Neither dose of BRL37344 modified neuronal density under hyperoxia (Figure 1).

In addition, hyperoxia also altered neuronal neurochemical coding.

**Figure 2:**
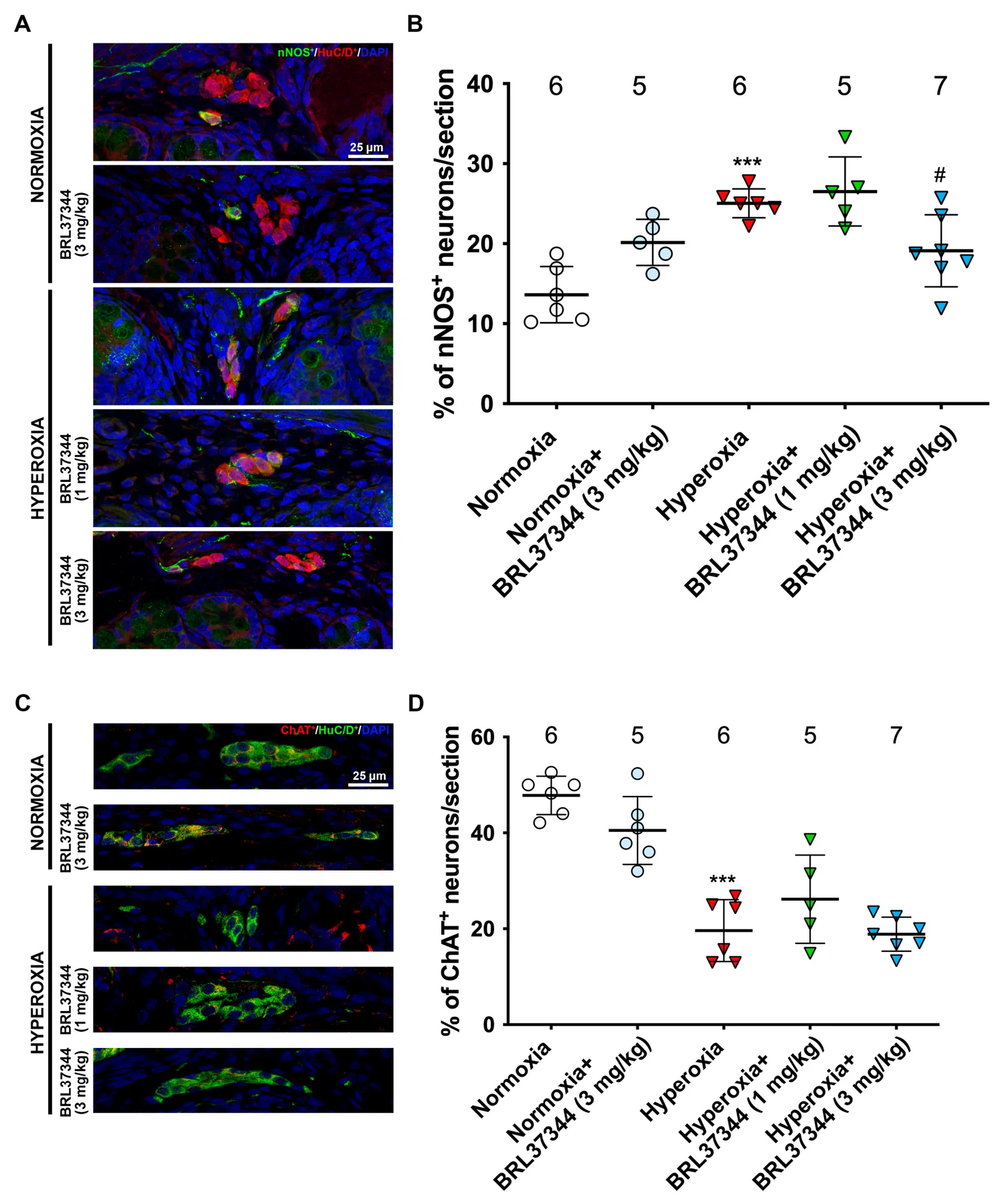
Hyperoxia alters the neurochemical coding of submucosal enteric neurons. Representative immunofluorescence images showing nNOS^+^ (green) and HuC/D^+^ (red) neurons (**A**), and ChAT^+^ (red) and HuC/D^+^ (green) neurons (**C**) within the colonic submucosal plexus. Nuclei were counterstained with DAPI (blue). Quantitative analysis demonstrated that neonatal hyperoxia significantly increased the proportion of nNOS^+^/HuC/D^+^ neurons (**B**) and reduced that of ChAT^+^ neurons (**D**) compared with the normoxic controls. BRL37344 at 3 mg/kg, but not 1 mg/kg, significantly reduced the hyperoxia-induced increase in the nitrergic neuronal population, whereas neither dose affected the cholinergic neuronal population. Data are presented as mean ± SD. Factorial analyses were performed by two-way ANOVA followed by Šídák’s test, and dose effects under hyperoxia by one-way ANOVA followed by Dunnett’s test. *** *p* < 0.001 vs. normoxia; ^#^ *p* < 0.05 vs. hyperoxia. Scale bar = 25 μm.

In particular, high oxygen levels increased the percentage of nNOS^+^ neurons compared with normoxic controls, whereas BRL37344 at 3 mg/kg, but not at 1 mg/kg, significantly reduced this hyperoxia-induced alteration (Figure 2B).

In contrast, hyperoxia significantly reduced the percentage of ChAT^+^ neurons (Figure 2C,D). This reduction was not modified by BRL37344 treatment, as indicated by the absence of a significant oxygen × treatment interaction (*p* = 0.1463) (Figure 2C,D).

### 3.2. Hyperoxia Differentially Affects Enteric Glial Markers in the Submucosal Plexus

To evaluate the enteric glial compartment of the submucosal plexus, the glia index was calculated as the SOX10^+^/HuC/D^+^ ratio, mirroring the relative abundance of enteric glial cells, whereas the S100β^+^/HuC/D^+^ ratio was used to estimate the differentiated enteric glial compartment.

**Figure 3:**
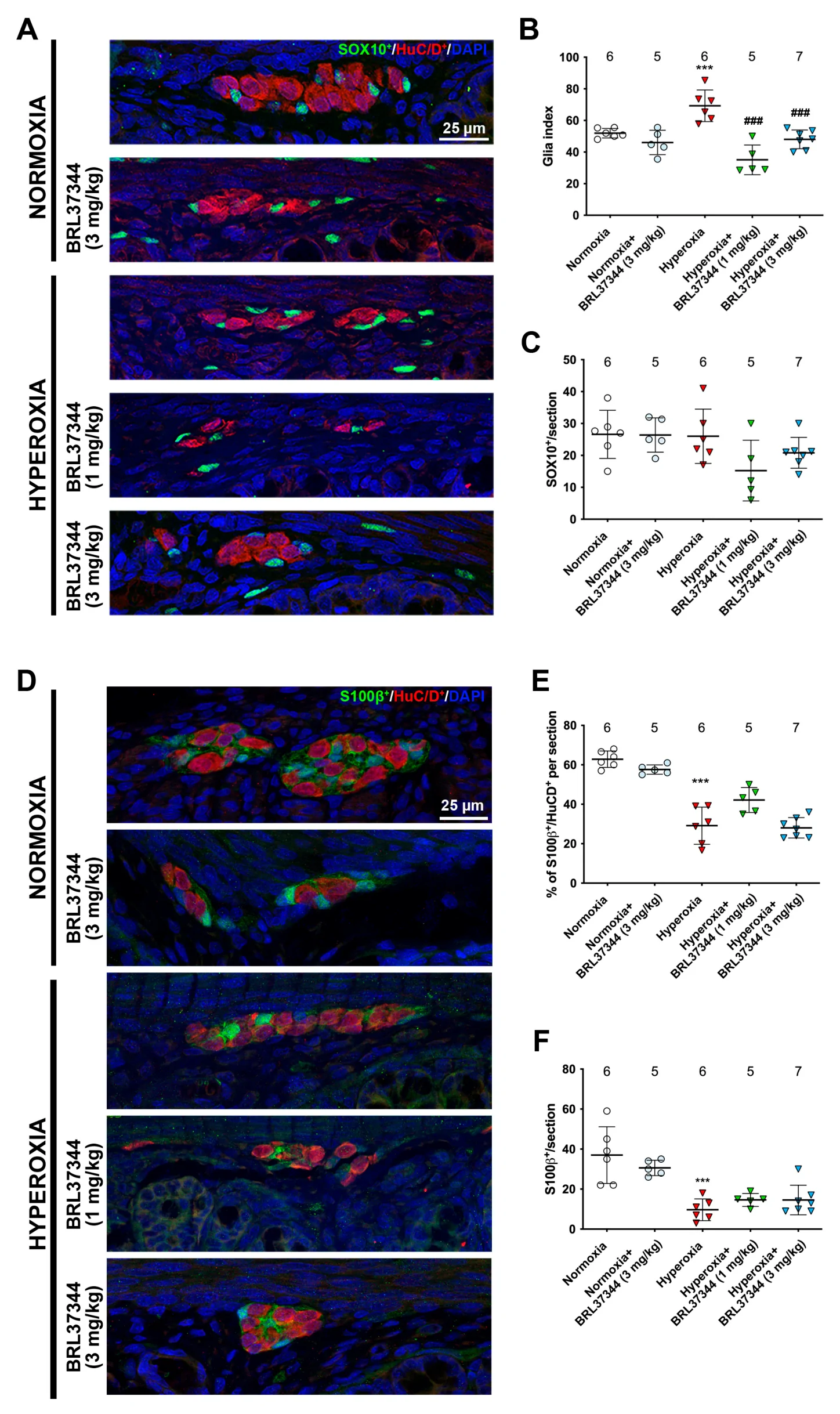
Hyperoxia differentially affects enteric glial populations and the neuron–glia balance in the colonic submucosal plexus. Representative immunofluorescence images and quantitative analyses of SOX10^+^ (green) and HuC/D^+^ (red) cells (**A**–**C**) and S100β^+^ (green) and HuC/D^+^ (red) cells (**D**–**F**) within the submucosal plexus. Nuclei were counterstained with DAPI (blue). Hyperoxia increased the SOX10^+^/HuC/D^+^ ratio (glia index) (**B**), whereas absolute SOX10^+^ cell counts did not significantly differ among experimental groups (**C**). BRL37344 at both 1 and 3 mg/kg reduced the hyperoxia-induced increase in glia index (**B**). In contrast, high oxygen levels significantly reduced both the S100β^+^/HuC/D^+^ ratio (**E**) and the absolute number of S100β^+^ cells (**F**), with no significant effect of BRL37344 treatment. Data are presented as mean ± SD. Factorial analyses were performed by two-way ANOVA followed by Šídák’s test, with dose effects under hyperoxic conditions assessed separately as described in the Statistical Analysis Section. *** *p* < 0.001 vs. normoxia; ^###^ *p* < 0.001 vs. hyperoxia. Scale bar = 25 μm.

Hyperoxic exposure significantly increased the glia index compared with normoxic controls (Figure 3A,B), while BRL37344 at both doses counteracted this alteration. Factorial analysis further showed that the effect of the *BRL37344* at 3 mg/kg was specific to hyperoxic conditions, as supported by a significant oxygen × treatment interaction (*p* = 0.0150), with no effect observed under normoxia. To determine whether the change in glia index reflected an actual increase in the glial population, absolute SOX10^+^ cell numbers were also evaluated. No significant differences were observed among experimental groups (Figure 3C), indicating that the observed change in SOX10^+^/HuC/D^+^ reflects an altered relative neuron–glia balance rather than an expansion of the SOX10^+^ glial population.

In contrast, hyperoxia significantly reduced the S100β^+^/HuC/D^+^ ratio, and this reduction persisted following BRL37344 treatment at either dose (Figure 3D,E). As for the glia index, we further examined whether this change was associated with an actual alteration in the glial population by assessing absolute S100β^+^ cell counts. Hyperoxia also significantly reduced the number of S100β^+^ cells, and BRL37344 treatment had no significant effect (Figure 3F). Together with the unchanged SOX10^+^ cell counts, these findings suggest that hyperoxia does not uniformly affect the overall enteric glial population, but preferentially involves the S100β^+^ glial compartment.

### 3.3. Hyperoxia Reduces the Mucosal SOX10^+^ Glial Cell Population in the Colonic Lamina Propria

**Figure 4:**
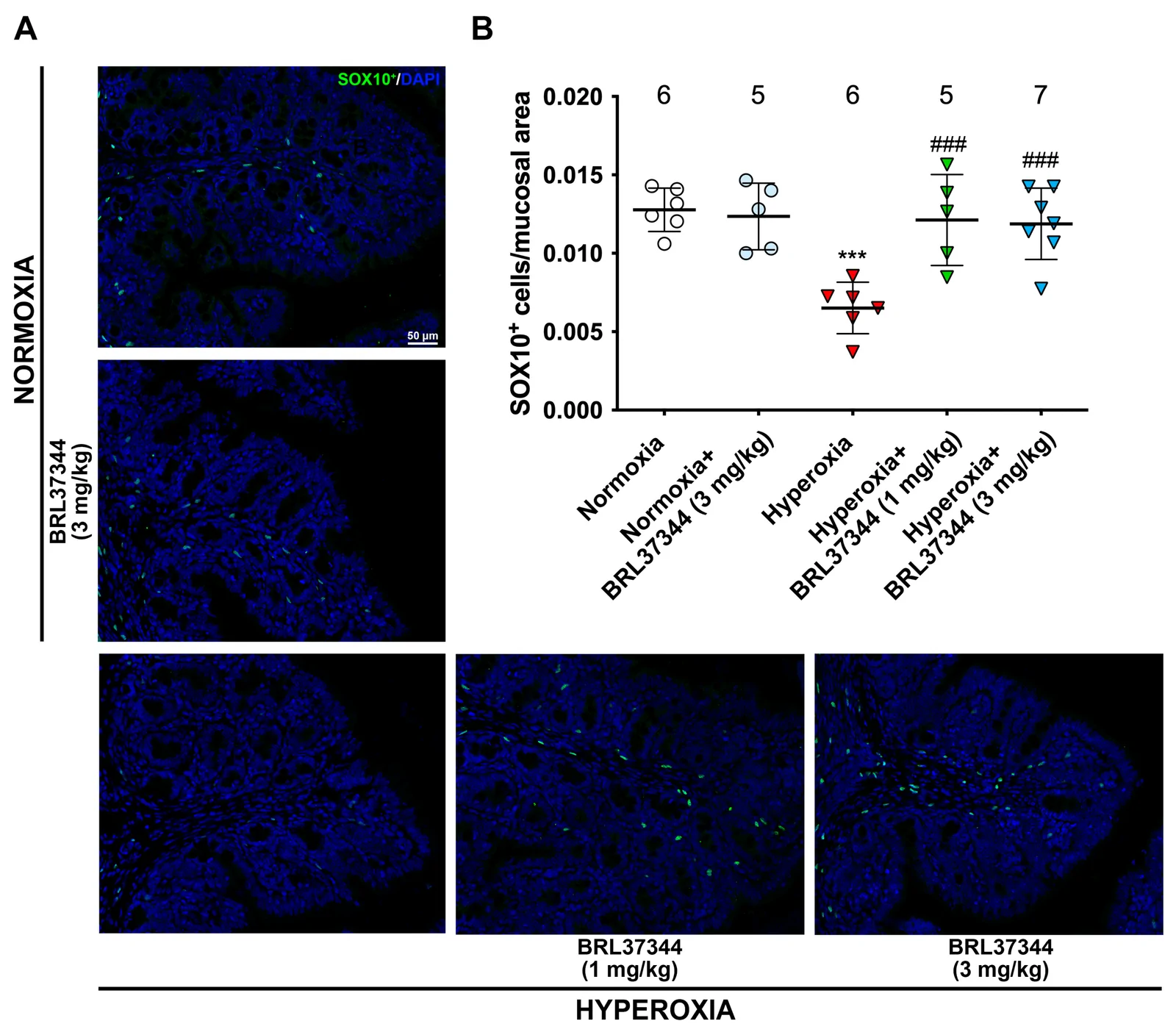
β3-Adrenergic receptor activation prevents hyperoxia-induced loss of SOX10^+^ mucosal enteric glial cells. Representative immunofluorescence images of SOX10^+^ cells (green) within the colonic lamina propria are shown in (**A**). Nuclei were counterstained with DAPI (blue). Quantitative morphometric analysis (**B**) demonstrated that neonatal hyperoxia significantly reduced the density of SOX10^+^ mucosal enteric glial cells compared with the normoxic controls. Treatment with BRL37344 at both doses prevented this reduction. No significant differences were observed between untreated and BRL37344-treated animals under normoxic conditions. Data are presented as mean ± SD. Factorial analyses were performed by two-way ANOVA followed by Šídák’s test, and dose effects under hyperoxia by one-way ANOVA followed by Dunnett’s test. *** *p* < 0.001 vs. normoxia; ^###^ *p* < 0.001 vs. hyperoxia. Scale bar = 50 μm.

To assess the effects of hyperoxia on the mucosal enteric glial compartment, SOX10-immunoreactive nuclei were quantified within the colonic lamina propria. Hyperoxic exposure significantly reduced the density of SOX10^+^ cells compared with normoxic controls, an effect that was prevented by BRL37344 at both doses (Figure 4A,B). Factorial analysis confirmed that the response to BRL37344 at 3 mg/kg depended on oxygen exposure (oxygen × treatment interaction, *p* = 0.0014), with no significant effect under normoxia (Figure 4A,B).

### 3.4. Hyperoxia-Associated Modifications in Relative Taxon Abundance of Colonic Microbiome

**Figure 5:**
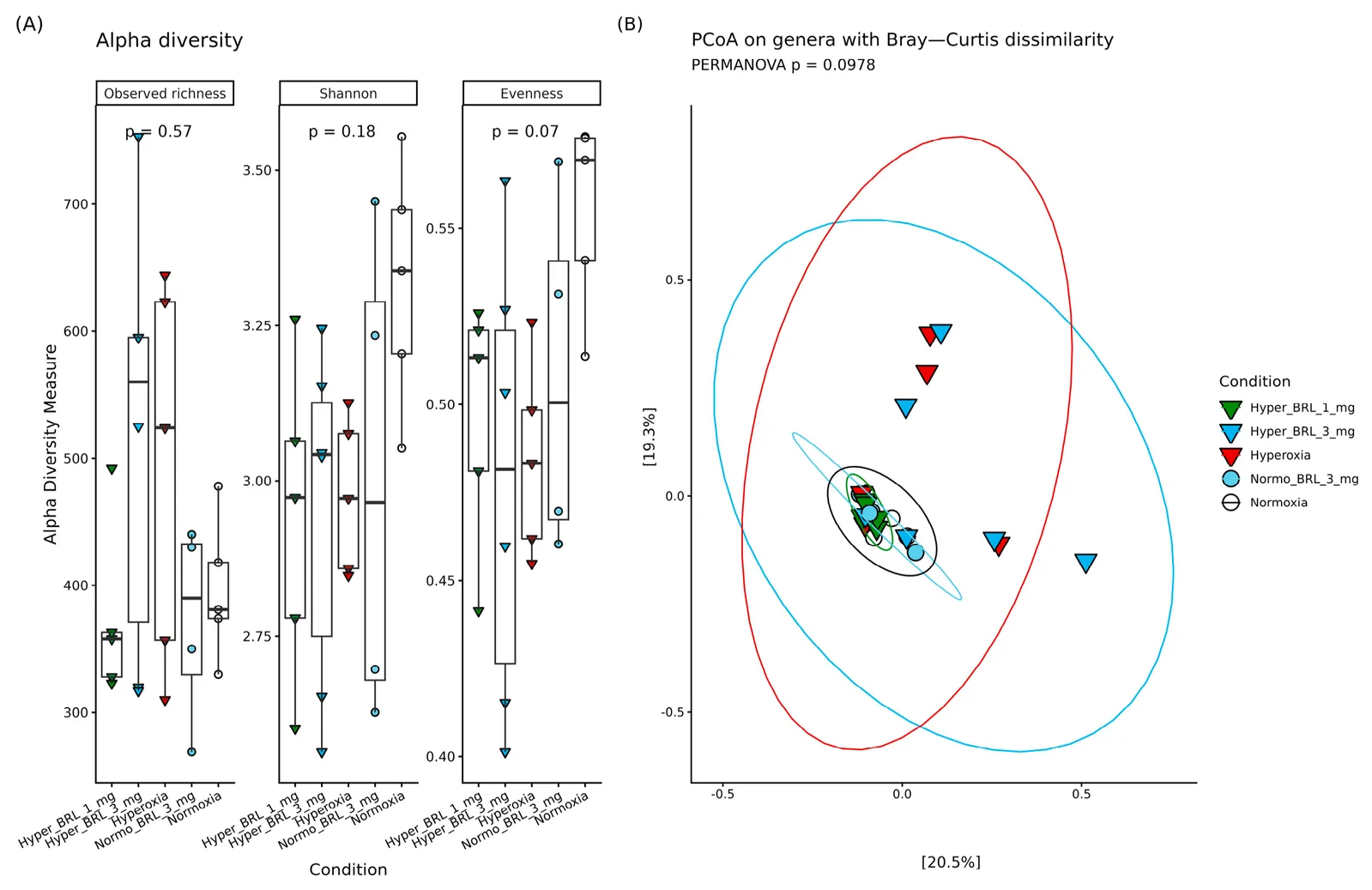
Neonatal hyperoxia does not alter the overall diversity or community structure of the colonic microbiome. Box plots showing α-diversity indices (**A**), including observed ASV richness, Shannon diversity, and Pielou’s evenness, across all experimental groups. No significant differences were detected among groups. Principal coordinates analysis (PCoA) (**B**) based on Hellinger distances calculated from genus-level relative abundances of colonic microbiome (CM) samples. Community structure did not differ significantly among the experimental groups. Data are presented as median ± IQR.

To investigate the effects of hyperoxia on the neonatal colonic microbiome, bacterial diversity, community structure, and taxonomic composition were analyzed by 16S rRNA gene sequencing. Following quality filtering, denoising, merging, chimera removal, and host-sequence filtering, 5,412,394 reads were retained across the analyzed samples, with a median sequencing depth of 144,121 reads per sample (Interquartile Range (IQR): 106,078–254,502). DADA2 processing generated 9793 ASVs, including 219 detected in the blank paraffin control. After contaminant and low-abundance filtering, 9203 ASVs were retained for downstream analyses. All samples passed the rarefaction curve quality control (Figure 5A). No significant differences in α-diversity were detected among the experimental groups, as assessed by observed richness, Shannon diversity and evenness indices (Figure 5A). Likewise, principal coordinates analysis (PCoA) based on Hellinger distances showed no significant differences in the overall microbial community structure between experimental groups (PERMANOVA, *p* = 0.184) (Figure 5B).

**Figure 6:**
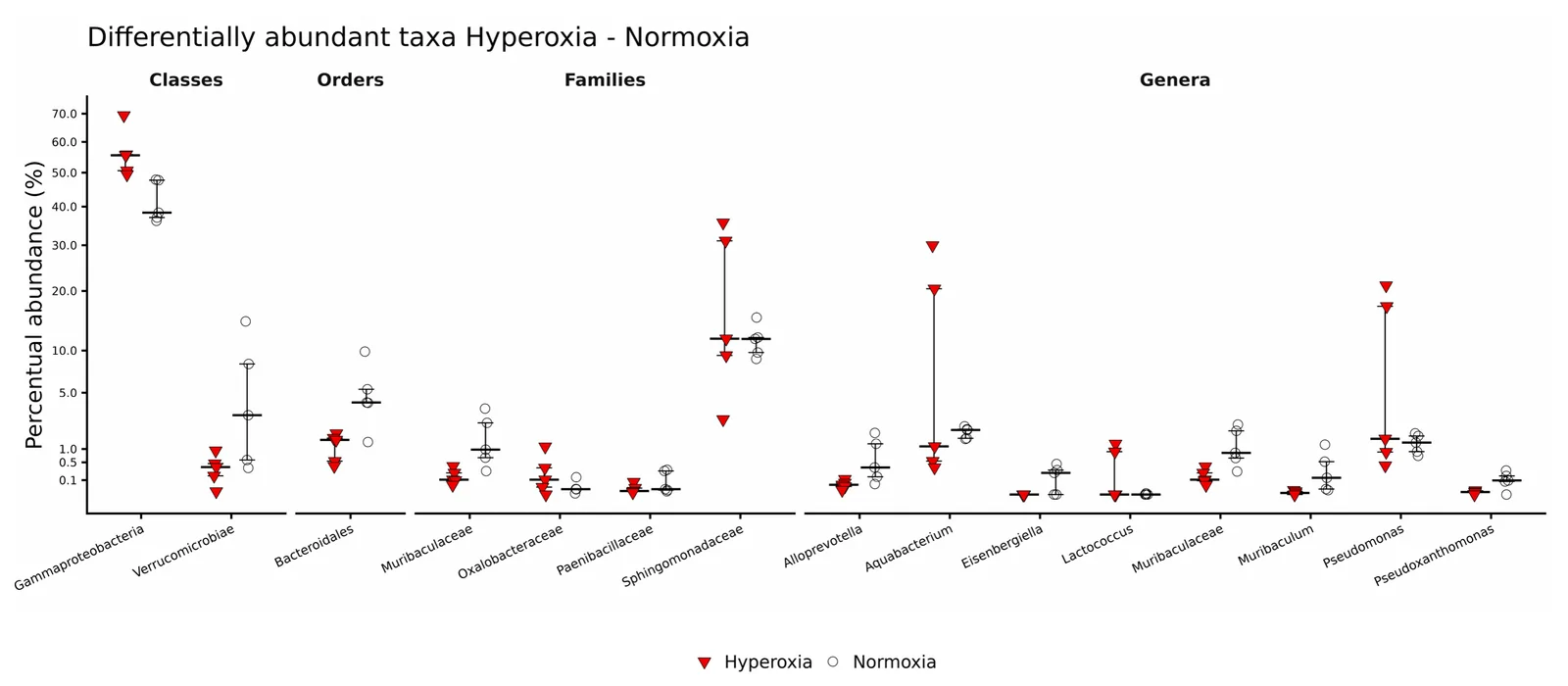
Neonatal hyperoxia selectively remodels the taxonomic composition of the colonic microbiome. Box plots showing bacterial taxa that were differentially abundant between normoxic and hyperoxic animals, as identified by differential abundance analysis. Only statistically significant taxa (adjusted *p* < 0.05) are shown. Data are presented as median ± IQR. Positive log_2_ fold-change values indicate taxa enriched in hyperoxic animals, whereas negative values indicate taxa depleted following hyperoxic exposure.

Despite the absence of significant differences in microbial diversity, differential abundance analysis revealed a selective remodeling of the neonatal CM associated with hyperoxic exposure (Figure 6). Compared with normoxic controls, hyperoxic exposure enriched bacterial taxa commonly associated with oxygen-tolerant and opportunistic microorganisms, including members of the class Gammaproteobacteria, the families Oxalobacteriaceae and Sphingomonadaceae, and the genera *Aquabacterium*, *Lactococcus*, and *Pseudomonas*. Conversely, bacterial groups associated with mucus utilization, complex carbohydrate metabolism and intestinal homeostasis, including members of the class Verrucomicrobiae, the order Bacteroidales, the family Muribaculaceae, and the genera *Muribaculum*, *Alloprevotella*, *Eisenbergiella* and *Pseudoxanthomonas*, were depleted following hyperoxic exposure.

**Figure 7:**
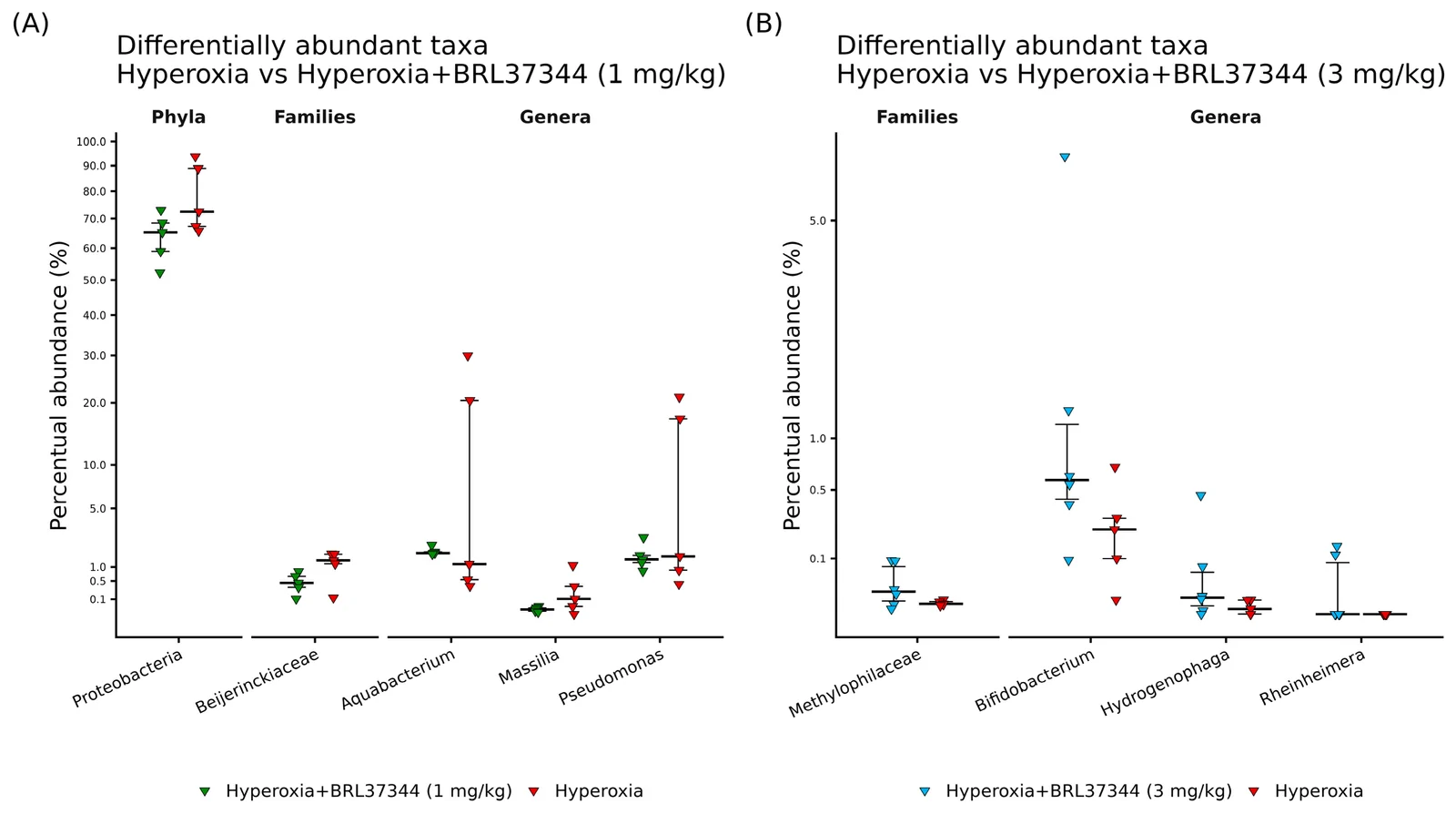
BRL37344 differentially modulates hyperoxia-induced microbial remodeling in a dose-dependent manner. (**A**) Differential abundance analysis comparing hyperoxic animals untreated or treated with BRL37344 (1 mg/kg). (**B**) Differential abundance analysis comparing hyperoxic animals untreated or treated with BRL37344 (3 mg/kg). Data are presented as median ± IQR. Only significant differentially abundant taxa (adjusted *p* < 0.05) are shown.

Compared with untreated hyperoxic animals, BRL37344 differentially modified the CM composition according to the administered dose. Treatment with BRL37344 at 1 mg/kg selectively reduced the abundance of several Proteobacteria-associated taxa, including members of the Proteobacteria phylum, the Beijerinckiaceae family and the genera *Massilia*, *Pseudomonas* and *Aquabacterium* (Figure 7A). In contrast, treatment with the 3 mg/kg dose selectively enriched a distinct subset of bacterial taxa, including members of the Methylophilaceae family and the neonatal-associated genus Bifidobacterium, as well as the genera *Hydrogenophaga* and *Rheinheimera* (Figure 7B).

**Figure 8:**
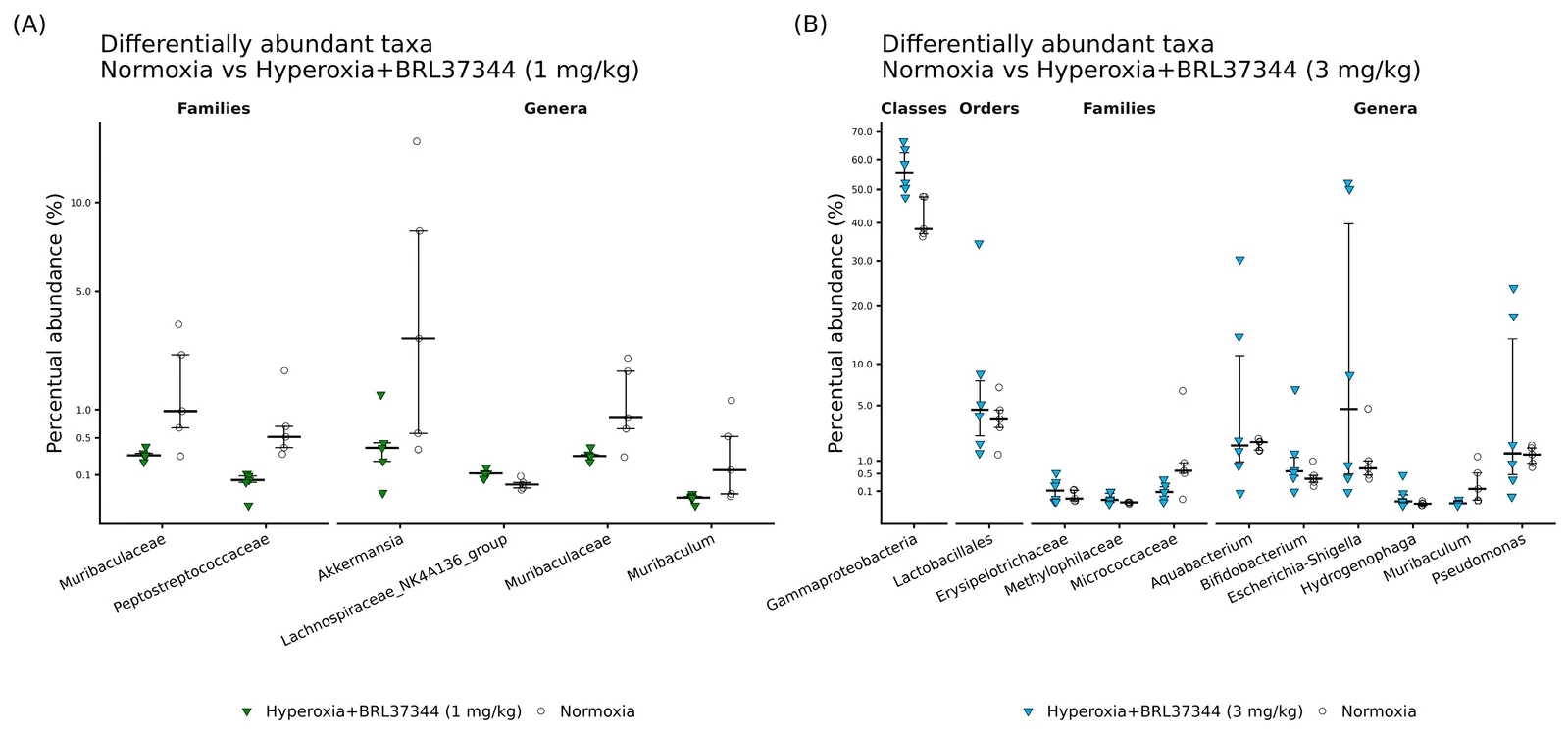
Colonic microbial profiles of BRL37344-treated hyperoxic animals remain distinct from normoxic controls. (**A**) Differential abundance analysis comparing normoxic animals with hyperoxic pups treated with BRL37344 (1 mg/kg). (**B**) Differential abundance analysis comparing normoxic animals with hyperoxic pups treated with BRL37344 (3 mg/kg). Positive and negative log_2_ fold-change values indicate taxa enriched or depleted, respectively, in BRL37344-treated hyperoxic animals relative to normoxic controls. Data are presented as median ± IQR. Only statistically significant taxa (adjusted *p* < 0.05) are shown.

Comparison with normoxic controls further showed that the CM profiles of hyperoxic groups treated with BRL37344 remained distinct from those observed under normoxic conditions. Compared with normoxic controls, pups receiving BRL37344 at 1 mg/kg exhibited lower abundances of several obligate anaerobe-associated taxa associated with mucus utilization and intestinal homeostasis, including members of the Muribaculaceae family and the genera *Muribaculum* and *Akkermansia*, whereas the Lachnospiraceae_NK4A136 group was enriched (Figure 8A). In contrast, the CM profile of pups treated with BRL37344 at 3 mg/kg remained characterized by enrichment of facultative anaerobic Enterobacteriaceae-related taxa, represented by *Escherichia-Shigella*, together with several Proteobacteria-associated genera, including *Pseudomonas, Aquabacterium,* and *Hydrogenophaga*. Finally, increased abundances of *Bifidobacterium* and members of the Lactobacillales order, Erysipelotrichaceae and Methylophilaceae families, were also observed, whereas *Muribaculum* and members of the Micrococcaceae family were depleted (Figure 8B).

**Figure 9:**
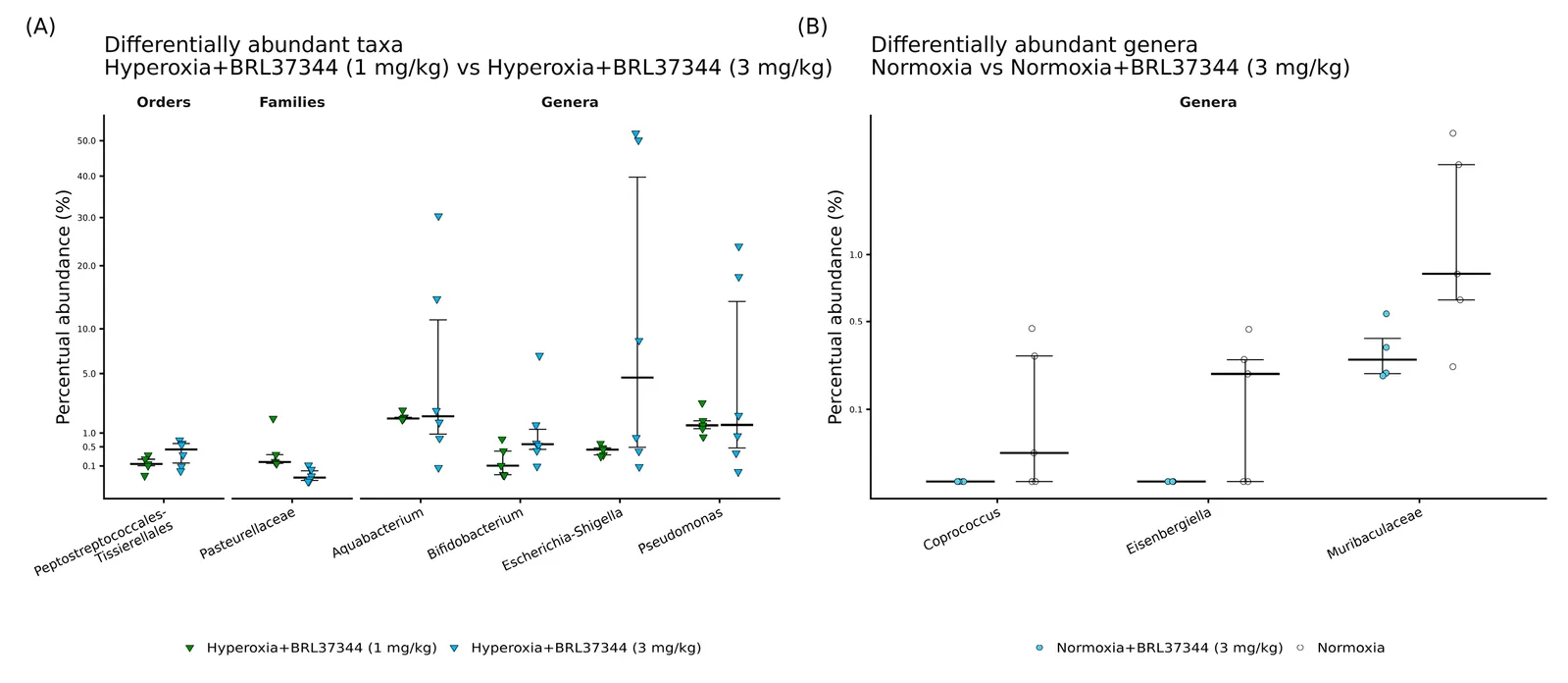
BRL37344 induces dose-dependent and oxygen-dependent changes in the colonic microbiome. (**A**) Differential abundance analysis comparing hyperoxic animals treated with BRL37344 at 1 mg/kg and 3 mg/kg. (**B**) Differential abundance analysis comparing normoxic animals untreated or treated with BRL37344 (3 mg/kg). Box plots show bacterial taxa that differed significantly between the compared groups. Positive and negative log_2_ fold-change values indicate taxa enriched or depleted, respectively, in the first group of each comparison. Data are presented as median ± IQR. Only statistically significant taxa (adjusted *p* < 0.05) are shown.

Direct comparison between the two BRL37344 doses under hyperoxic conditions further demonstrated that each treatment generated a distinct microbial signature (Figure 9A). Compared with the lower dose, treatment with 3 mg/kg of BRL37344 was associated with enrichment of members of the Peptostreptococcales-Tissierellales order and the genera *Bifidobacterium*, *Aquabacterium*, *Escherichia-Shigella*, and *Pseudomonas*, whereas members of the Pasteurellaceae family were more abundant in animals receiving the 1 mg/kg dose (Figure 9A). Under normoxic conditions, BRL37344 exerted only modest effects on the colonic microbiome. Compared with untreated normoxic animals, BRL37344-treated pups showed a reduction in the *genera Coprococcus* and *Eisenbergiella*, together with an unclassified member of the Muribaculaceae family (Figure 9B).

## 4. Discussion

The present study identifies the enteric nervous system and the CM as two major developmental targets of neonatal hyperoxia in the colon. Under physiological conditions, these systems undergo coordinated maturation and reciprocal interactions that are essential for postnatal intestinal development [29,30]. To our knowledge, this is the first study that simultaneously investigates, within the same experimental model, the effects of postnatal hyperoxia on the mucosal and submucosal compartments of the enteric nervous system and on the composition of the CM. The selective pattern of modulation observed following BRL37344 treatment suggests a possible involvement of β3-AR signaling in the response of the developing intestine to altered oxygen exposure and raises the hypothesis that this pathway may contribute to oxygen-dependent postnatal intestinal adaptation.

The neonatal colon is particularly vulnerable to alterations in oxygen availability because a relevant part of its structural and functional maturation occurs after birth [31]. Normal postnatal intestinal maturation critically depends on tightly regulated oxygen homeostasis, and deviations in either direction, including hypoxia and hyperoxia, perturb coordinated developmental processes [32,33,34]. In particular, neonatal hyperoxia has been shown to impair proximal colonic maturation by compromising epithelial barrier integrity [35], reducing mucin production and the number of β3-AR-expressing cells, and altering the neurochemical coding of the myenteric plexus [7]. Additionally, similar alterations have also been described in the ileum, where neonatal hyperoxia impairs epithelial development [17,36], compromises barrier integrity [5,37], disrupts vascularization [13], and alters the enteric nervous system [14]. Moreover, neonatal exposure to high oxygen levels has been shown to selectively remodel the intestinal microbiome [27,28,34], further supporting the concept that physiological oxygen homeostasis is essential for coordinated postnatal intestinal maturation.

This study extends our previous observations on the myenteric plexus in a similar animal model to the submucosal plexus and to the mucosal enteric glia of the proximal colon. Hyperoxia exposure induced a marked loss of submucosal neurons together with neurochemical remodeling, characterized by an increased proportion of nitrergic neurons and a concomitant reduction in cholinergic neurons. These findings are consistent with our previous observations in the submucosal plexus of the ileum [14], indicating that the pattern of hyperoxia-induced submucosal remodeling is conserved across intestinal regions. In contrast, they differ markedly from the response previously observed in the myenteric plexus of both the ileum and the proximal colon, where hyperoxia did not affect overall neuronal density and induced the opposite pattern of neurochemical remodeling, characterized by reduced nitrergic and increased cholinergic neurons [7,13]. The opposite responses observed in the myenteric and submucosal plexuses can be explained by the distinct developmental and functional organization of these two enteric compartments. Recent studies have demonstrated that the myenteric and submucosal plexuses represent anatomically, developmentally, and functionally distinct compartments of the enteric nervous system, characterized by different maturation programs, neuronal connectivity, and physiological specialization [38,39,40].

The glial alterations observed in the present study further demonstrate that neonatal hyperoxia impairs maturation of the enteric nervous system. Within the submucosal plexus, hyperoxia increased the glia index (expressed as the SOX10^+^/HuC/D^+^ ratio) while leaving the absolute number of SOX10^+^ cells unchanged, indicating that the increased ratio reflects an altered neuron–glia balance rather than an expansion of the SOX10+ glial population. In contrast, the reduction in the S100β^+^/HuC/D^+^ ratio was accompanied by a significant decrease in the absolute number of S100β+ cells. These findings indicate that oxygen imbalance alters the physiological organization of enteric glia during postnatal maturation, not uniformly affecting the enteric glial population but preferentially involving the differentiated S100β^+^ glial compartment.

Interestingly, BRL37344 treatment reduced the hyperoxia-induced increase in glia index without affecting the number of SOX10^+^ cells, suggesting a potential effect on the relative neuron–glia organization rather than on glial abundance. Moreover, the BRL37344 treatment did not prevent the hyperoxia-induced reduction in the S100β^+^/HuC/D^+^ ratio nor the absolute number of S100β^+^ cells. These findings are consistent with the neuronal localization of β3-AR and the absence of receptor expression in enteric glia [14,41]. Although an indirect influence of β3-AR on neuron–glia interactions may be hypothesized, the present study does not allow the underlying mechanism or receptor-specific causality to be established.

The effects of hyperoxia on the enteric glial compartment were not confined to the submucosal plexus. Neonatal hyperoxia also reduced the presence of SOX10^+^ cells within the colonic lamina propria. This observation is particularly relevant because recent studies have demonstrated that colonization of the intestinal mucosa by enteric glial cells critically depends on the gut microbiome [26,29]. Although the present study does not establish a causal relationship, the concomitant hyperoxia-induced reduction in SOX10^+^ mucosal glial cells together with selective microbial remodeling are consistent with the proposed mucosal glial developmental model and suggest that high oxygen levels simultaneously perturb these two tightly interconnected components of postnatal intestinal maturation [26].

Interestingly, β3-AR activation prevented the hyperoxia-induced reduction in SOX10^+^ glial cells in the mucosa and was also associated with selective remodeling of the CM. Whether these effects are mechanistically related remains to be elucidated. However, evidence that the adrenergic system participates in bidirectional communication between the host and the intestinal microbiome [42] provides a biological framework for interpreting these concomitant effects. Within this context, the parallel changes observed in the mucosal glial compartment and CM following BRL37344 treatment raise the possibility of an interplay among adrenergic signaling, enteric glia, and the gut microbiome during neonatal hyperoxia. The present experimental design, however, does not establish a causal or mechanistic relationship among these components, which will require direct experimental investigation.

The alterations observed in the mucosal glial compartment were accompanied by a selective remodeling of the developing CM. However, neither α- nor β-diversity differed significantly among experimental groups, indicating that the observed changes did not reflect a global restructuring of the microbial community but rather involved specific bacterial taxa. In particular, oxygen imbalance was associated with a shift from obligate anaerobic bacteria linked to mucus utilization and epithelial homeostasis, including members of the Muribaculaceae family and the genus *Akkermansia* [43], towards facultative and oxygen-tolerant microorganisms, such as members of the *Gammaproteobacteria* class [44].

The depletion of Muribaculaceae and *Akkermansia* is consistent with our previous observations demonstrating impaired mucin production and delayed epithelial maturation following neonatal hyperoxia [7], suggesting that oxygen imbalance simultaneously disrupts both the epithelial niche and the microbial communities that normally support gut barrier maturation. Conversely, expansion of Gammaproteobacteria aligns with previous studies describing selective enrichment of facultative and oxygen-tolerant microorganisms following neonatal hyperoxia [17,28]. Because establishment of an anaerobic intestinal environment is a prerequisite for physiological postnatal microbial assembly and mucosal maturation [45], these taxon-specific changes suggest that hyperoxia may influence the normal developmental trajectory of the CM without inducing a generalized disruption of microbial community structure.

The effects of β3-AR activation on the intestinal microbiome followed an unexpected pattern, showing distinct dose-dependent microbial configurations. The lower dose predominantly attenuated the hyperoxia-associated expansion of facultative and oxygen-tolerant taxa, including *Pseudomonas* and *Aquabacterium*, suggesting attenuation of some hyperoxia-associated taxonomic changes. Conversely, the higher dose did not restore the normoxic microbial profile but promoted enrichment of *Bifidobacterium*, a bacterial genus widely recognized as one of the principal earliest colonizers of the mammalian intestine and a key contributor to physiological microbial assembly in newborns [46,47,48]. The observation that β3-AR activation increased *Bifidobacterium* abundance even under normoxic conditions suggests that its effects extend beyond protection from hyperoxia and may involve modulation of other developmental host–microbiome interactions.

This finding is particularly interesting because a similar microbial signature, characterized by depletion of *Bifidobacterium* together with expansion of Proteobacteria, has been described in preterm infants before the onset of necrotizing enterocolitis [49,50], indicating that these microbial alterations precede overt intestinal disease. A comparable microbial profile has also been associated with bronchopulmonary dysplasia [51], another neonatal disorder strongly linked to anticipated, prolonged hyperoxic exposure. Notably, we recently demonstrated that the same β3-AR agonist, BRL37344, at the dose of 3 mg/kg effectively counteracted experimental bronchopulmonary dysplasia [15]. Although these observations do not establish a causal relationship, they suggest that β3-AR signaling may preserve coordinated developmental trajectories rather than simply counteracting established tissue injury. Consequently, the greatest biological and potentially therapeutic relevance of β3-AR activation may lie during the early phases of neonatal adaptation, when developmental programs remain plastic, before inflammatory cascades become established and tissue injury becomes self-sustaining.

Several limitations should be considered when interpreting the present findings.

First, BRL37344 was selected as the β3-AR agonist because its pharmacological profile and dose–response relationship have been extensively characterized in previous experimental studies [7,13,14,15], allowing direct comparison with earlier findings and ensuring continuity across the experimental models. Moreover, although BRL37344 is commonly used as a β3-AR agonist, pharmacological agonism alone does not establish receptor-specific causality. Therefore, while the observed effects are consistent with the involvement of β3-AR signaling, confirmation of a direct receptor-mediated mechanism will require complementary approaches, including receptor antagonism or genetic manipulation.

From a translational perspective, further studies are needed to evaluate whether mirabegron, the only β3-AR agonist currently approved for clinical use, also maintains similar effects. Given the different pharmacokinetic profile of mirabegron, dedicated dose-finding and treatment-schedule studies in animal models of prematurity-associated diseases are mandatory. This aspect is particularly relevant in newborns, more susceptible to metabolic and growth imbalances. Defining an appropriate therapeutic window will therefore be essential to preserve the developmental benefits of β3-AR activation while minimizing unwanted side effects, particularly the well-known metabolic/lipolytic effects.

Second, although our study has shown that neonatal hyperoxia induced concomitant alterations in the colonic microbiome and the mucosal enteric glial compartment, the reported data are insufficient to establish a direct causal relationship between these events. Future studies combining controlled microbiome manipulation with detailed characterization of enteric glial development will be necessary to determine whether microbial alterations directly contribute to the observed glial phenotype.

Finally, microbiome analyses were performed on formalin-fixed paraffin-embedded colonic tissue samples. Although this approach enabled direct correlation between histological and microbiome analyses within the same specimens, formalin fixation, DNA degradation, and the relatively low microbial biomass in these samples may have reduced DNA yield and taxonomic resolution. Future studies using fresh intestinal samples and shotgun metagenomic sequencing will be required to validate and extend the present findings.

Addressing these limitations will contribute to clarifying the role of β3-AR signaling in intestinal development and facilitate translation of the present findings into clinically relevant therapeutic protocols in neonatology.

## 5. Conclusions

Neonatal hyperoxia profoundly alters the coordinated postnatal maturation of the proximal colon, affecting the submucosal and mucosal enteric nervous system and the CM. Our findings demonstrate that treatment with the BRL37344 selectively modulates these hyperoxia-induced alterations, modulating neuron–glia organization within the submucosal plexus, preventing the loss of mucosal enteric glia, and being associated with distinct developmental changes in the intestinal microbiome. Together, these observations support the hypothesis that β3-AR signaling contributes to physiological intestinal adaptation during the early postnatal period and provide a rationale for exploring β3-AR agonism as a possible therapeutic strategy to preserve intestinal development in neonatal conditions associated with oxygen imbalance.

## Figures and Tables

**Table 1:** Primary and secondary antisera used in immunofluorescence analysis.

Antigen	Species	Source	Catalog Number	Concentration
**Primary Antisera**				
ChAT	Goat	Merck, Darmstadt, Germany	AB144P	1:200
nNOS	Rabbit	GeneTex (Irvine, CA, USA)	GTX133403	1:500
S100β	Rabbit	Genetex	GTX129573	1:500
SOX10	Rabbit	Abcam	Ab227680	1:50
HuC/D	Mouse	Invitrogen (Waltham, MA, USA)	A21271	1:200
**Secondary Antisera**				
Alexa Fluor 488	Donkey	Jackson ImmunoResearch (Ely, UK)	711-545-152	1:175
Alexa Fluor 488	Donkey	Jackson ImmunoResearch	703-545-155	1:175
Alexa Fluor 594	Donkey	Jackson ImmunoResearch	711-585-152	1:175
Alexa Fluor 594	Sheep	Jackson ImmunoResearch	515-585-062	1:175
Alexa Fluor 488	Sheep	Jackson ImmunoResearch	515-545-062	1:175
Alexa Fluor 594	Bovine	Jackson ImmunoResearch	805-585-180	1:175

## Data Availability

The data presented in this study are deposited in the NCBI Gene Expression Omnibus (GEO) repository, accession number GSE344801.

## Abbreviations

The following abbreviations are used in this manuscript:

ANOVA	one-way analysis of variance
ASVs	amplicon sequence variants
BPD	bronchopulmonary dysplasia
BSA	bovine serum albumin
ChAT	choline acetyltransferase
CM	colonic microbiome
Crl:CD(SD)	Sprague–Dawley rats
DAPI	4′,6-diamidino-2-phenylindole
ENS	enteric nervous system
FFPE	Formalin-Fixed Paraffin-Embedded
MICCA	MICrobial Community Analysis
NEC	necrotising enterocolitis
nNOS	neuronal nitric oxide synthase
PBS	phosphate-buffered saline
PCoA	principal coordinate analysis
PERMANOVA	permutational multivariate analysis of variance
PFA	paraformaldehyde
ROP	retinopathy of prematurity
SD	standard deviation
SOX10	SRY-box transcription factor 10
β3-AR	β3-adrenergic receptor

## References

[1] Simon M.C., Keith B. (2008). The Role of Oxygen Availability in Embryonic Development and Stem Cell Function. Nat. Rev. Mol. Cell Biol..

[2] Fathollahipour S., Patil P.S., Leipzig N.D. (2018). Oxygen Regulation in Development: Lessons from Embryogenesis Towards Tissue Engineering. Cells Tissues Organs.

[3] Coelho-Santos V., Shih A.Y. (2020). Postnatal Development of Cerebrovascular Structure and the Neurogliovascular Unit. Wiley Interdiscip. Rev. Dev. Biol..

[4] O’Reilly M., Thébaud B. (2014). Animal Models of Bronchopulmonary Dysplasia. The Term Rat Models. Am. J. Physiol.-Lung Cell. Mol. Physiol..

[5] Chou H.-C., Chen C.-M. (2017). Neonatal Hyperoxia Disrupts the Intestinal Barrier and Impairs Intestinal Function in Rats. Exp. Mol. Pathol..

[6] Filippi L., Pini A., Cammalleri M., Bagnoli P., Dal Monte M. (2022). Β3-Adrenoceptor, a Novel Player in the Round-Trip from Neonatal Diseases to Cancer: Suggestive Clues from Embryo. Med. Res. Rev..

[7] Filippi L., Nardini P., Zizi V., Molino M., Fazi C., Calvani M., Carrozzo F., Cavallaro G., Giuseppetti G., Calosi L. (2023). Β3 Adrenoceptor Agonism Prevents Hyperoxia-Induced Colonic Alterations. Biomolecules.

[8] Schena G., Caplan M.J. (2019). Everything You Always Wanted to Know About β(3)-AR * (* But Were Afraid to Ask). Cells.

[9] Fujinaga M., Scott J.C. (1997). Gene Expression of Catecholamine Synthesizing Enzymes and Beta Adrenoceptor Subtypes during Rat Embryogenesis. Neurosci. Lett..

[10] Filippi L., Cammalleri M., Amato R., Ciantelli M., Pini A., Bagnoli P., Dal Monte M. (2022). Decoupling Oxygen Tension From Retinal Vascularization as a New Perspective for Management of Retinopathy of Prematurity. New Opportunities From β-Adrenoceptors. Front. Pharmacol..

[11] Filippi L., Scaramuzzo R.T., Pascarella F., Pini A., Morganti R., Cammalleri M., Bagnoli P., Ciantelli M. (2023). Fetal Oxygenation in the Last Weeks of Pregnancy Evaluated Through the Umbilical Cord Blood Gas Analysis. Front. Pediatr..

[12] Dal Monte M., Filippi L., Bagnoli P. (2013). Beta3-Adrenergic Receptors Modulate Vascular Endothelial Growth Factor Release in Response to Hypoxia Through the Nitric Oxide Pathway in Mouse Retinal Explants. Naunyn-Schmiedeberg’s Arch. Pharmacol..

[13] Nardini P., Zizi V., Molino M., Fazi C., Calvani M., Carrozzo F., Giuseppetti G., Calosi L., Guasti D., Biagini D. (2024). Protective Effects of Beta-3 Adrenoceptor Agonism on Mucosal Integrity in Hyperoxia-Induced Ileal Alterations. Antioxidants.

[14] Nardini P., Filippi L., Zizi V., Molino M., Fazi C., Chivetti M., Pini A. (2025). Beta-3 Adrenoceptor Agonism Protects the Enteric Nervous Tissue Against Hyperoxia-Induced Damage. Cells.

[15] Pini A., Nardini P., Zizi V., Molino M., Calvani M., Carrozzo F., Amato R., Guasti D., Biagini D., di Francesco F. (2026). β(3)-Adrenoceptor Agonism Exerts Lung Protection in a Rat Model of Bronchopulmonary Dysplasia. Br. J. Pharmacol..

[16] Li T.-M., Liu D.-Y. (2022). Mechanism of Neonatal Intestinal Injury Induced by Hyperoxia Therapy. J. Immunol. Res..

[17] Wang H.-C., Chou H.-C., Chen C.-M. (2023). Molecular Mechanisms of Hyperoxia-Induced Neonatal Intestinal Injury. Int. J. Mol. Sci..

[18] Vergnolle N., Cirillo C. (2018). Neurons and Glia in the Enteric Nervous System and Epithelial Barrier Function. Physiology.

[19] Pawolski V., Schmidt M.H.H. (2020). Neuron-Glia Interaction in the Developing and Adult Enteric Nervous System. Cells.

[20] Arrieta M.-C., Stiemsma L.T., Amenyogbe N., Brown E.M., Finlay B. (2014). The Intestinal Microbiome in Early Life: Health and Disease. Front. Immunol..

[21] Sanidad K.Z., Zeng M.Y. (2020). Neonatal Gut Microbiome and Immunity. Curr. Opin. Microbiol..

[22] Zhang H., Zhang Z., Liao Y., Zhang W., Tang D. (2022). The Complex Link and Disease Between the Gut Microbiome and the Immune System in Infants. Front. Cell. Infect. Microbiol..

[23] Yang I., Corwin E.J., Brennan P.A., Jordan S., Murphy J.R., Dunlop A. (2016). The Infant Microbiome: Implications for Infant Health and Neurocognitive Development. Nurs. Res..

[24] Underwood M.A., Mukhopadhyay S., Lakshminrusimha S., Bevins C.L. (2020). Neonatal Intestinal Dysbiosis. J. Perinatol..

[25] Hung L.Y., Parathan P., Boonma P., Wu Q., Wang Y., Haag A., Luna R.A., Bornstein J.C., Savidge T.C., Foong J.P.P. (2020). Antibiotic Exposure Postweaning Disrupts the Neurochemistry and Function of Enteric Neurons Mediating Colonic Motor Activity. Am. J. Physiol. Gastrointest. Liver Physiol..

[26] Kabouridis P.S., Lasrado R., McCallum S., Chng S.H., Snippert H.J., Clevers H., Pettersson S., Pachnis V. (2015). Microbiota Controls the Homeostasis of Glial Cells in the Gut Lamina Propria. Neuron.

[27] Lo Y.-C., Chen K.-Y., Chou H.-C., Lin I.-H., Chen C.-M. (2021). Neonatal Hyperoxia Induces Gut Dysbiosis and Behavioral Changes in Adolescent Mice. J. Chin. Med. Assoc..

[28] Li Y., Tao Y., Xu J., He Y., Zhang W., Jiang Z., He Y., Liu H., Chen M., Zhang W. (2021). Hyperoxia Provokes Time- and Dose-Dependent Gut Injury and Endotoxemia and Alters Gut Microbiome and Transcriptome in Mice. Front. Med..

[29] Obata Y., Pachnis V. (2016). The Effect of Microbiota and the Immune System on the Development and Organization of the Enteric Nervous System. Gastroenterology.

[30] Obata Y., Castaño Á., Boeing S., Bon-Frauches A.C., Fung C., Fallesen T., de Agüero M.G., Yilmaz B., Lopes R., Huseynova A. (2020). Neuronal Programming by Microbiota Regulates Intestinal Physiology. Nature.

[31] Henning S.J. (1981). Postnatal Development: Coordination of Feeding, Digestion, and Metabolism. Am. J. Physiol.-Gastrointest. Liver Physiol..

[32] Dotinga B.M., Mintzer J.P., Moore J.E., Hulscher J.B.F., Bos A.F., Kooi E.M.W. (2020). Maturation of Intestinal Oxygenation: A Review of Mechanisms and Clinical Implications for Preterm Neonates. Front. Pediatr..

[33] D’Souza A., Cai C.L., Kumar D., Cai F., Fordjour L., Ahmad A., Valencia G., Aranda J.V., Beharry K.D. (2012). Cytokines and Toll-like Receptor Signaling Pathways in the Terminal Ileum of Hypoxic/Hyperoxic Neonatal Rats: Benefits of Probiotics Supplementation. Am. J. Transl. Res..

[34] Latkowska M., Cai C.L., Mitrou M., Marcelino M., Aranda J.V., Beharry K.D. (2025). Gut Microbiome and Inflammation in Response to Increasing Intermittent Hypoxia in the Neonatal Rat. Pediatr. Res..

[35] Liu D.Y., Lou W.J., Zhang D.Y., Sun S.Y. (2020). ROS Plays a Role in the Neonatal Rat Intestinal Barrier Damages Induced by Hyperoxia. BioMed Res. Int..

[36] Giannone P.J., Bauer J.A., Schanbacher B.L., Reber K.M. (2007). Effects of Hyperoxia on Postnatal Intestinal Development. Biotech. Histochem..

[37] Chen C.-M., Chou H.-C. (2016). Hyperoxia Disrupts the Intestinal Barrier in Newborn Rats. Exp. Mol. Pathol..

[38] Li Z., Hao M.M., Van den Haute C., Baekelandt V., Boesmans W., Vanden Berghe P. (2019). Regional Complexity in Enteric Neuron Wiring Reflects Diversity of Motility Patterns in the Mouse Large Intestine. Elife.

[39] Hamnett R., Dershowitz L.B., Sampathkumar V., Wang Z., Gomez-Frittelli J., De Andrade V., Kasthuri N., Druckmann S., Kaltschmidt J.A. (2022). Regional Cytoarchitecture of the Adult and Developing Mouse Enteric Nervous System. Curr. Biol..

[40] Sharkey K.A., Mawe G.M. (2023). The Enteric Nervous System. Physiol. Rev..

[41] Cellek S., Thangiah R., Bassil A.K., Campbell C.A., Gray K.M., Stretton J.L., Lalude O., Vivekanandan S., Wheeldon A., Winchester W.J. (2007). Demonstration of Functional Neuronal Beta3-Adrenoceptors within the Enteric Nervous System. Gastroenterology.

[42] Rahmdel S., Luqman A., Götz F. (2025). Microbiota-Derived Aromatic Amino Acid Decarboxylases: Linking Microbial Fitness and Host Neurochemical Communication. mBio.

[43] Zhu Y., Chen B., Zhang X., Akbar M.T., Wu T., Zhang Y., Zhi L., Shen Q. (2024). Exploration of the Muribaculaceae Family in the Gut Microbiota: Diversity, Metabolism, and Function. Nutrients.

[44] Albenberg L., Esipova T.V., Judge C.P., Bittinger K., Chen J., Laughlin A., Grunberg S., Baldassano R.N., Lewis J.D., Li H. (2014). Correlation between Intraluminal Oxygen Gradient and Radial Partitioning of Intestinal Microbiota. Gastroenterology.

[45] Nicholson J.K., Holmes E., Kinross J., Burcelin R., Gibson G., Jia W., Pettersson S. (2012). Host-Gut Microbiota Metabolic Interactions. Science.

[46] Underwood M.A., German J.B., Lebrilla C.B., Mills D.A. (2015). Bifidobacterium Longum Subspecies Infantis: Champion Colonizer of the Infant Gut. Pediatr. Res..

[47] Stewart C.J., Ajami N.J., O’Brien J.L., Hutchinson D.S., Smith D.P., Wong M.C., Ross M.C., Lloyd R.E., Doddapaneni H., Metcalf G.A. (2018). Temporal Development of the Gut Microbiome in Early Childhood from the TEDDY Study. Nature.

[48] Henrick B.M., Rodriguez L., Lakshmikanth T., Pou C., Henckel E., Arzoomand A., Olin A., Wang J., Mikes J., Tan Z. (2021). Bifidobacteria-Mediated Immune System Imprinting Early in Life. Cell.

[49] Pammi M., Cope J., Tarr P.I., Warner B.B., Morrow A.L., Mai V., Gregory K.E., Kroll J.S., McMurtry V., Ferris M.J. (2017). Intestinal Dysbiosis in Preterm Infants Preceding Necrotizing Enterocolitis: A Systematic Review and Meta-Analysis. Microbiome.

[50] Masi A.C., Embleton N.D., Lamb C.A., Young G., Granger C.L., Najera J., Smith D.P., Hoffman K.L., Petrosino J.F., Bode L. (2021). Human Milk Oligosaccharide DSLNT and Gut Microbiome in Preterm Infants Predicts Necrotising Enterocolitis. Gut.

[51] Thatrimontrichai A., Praditaukrit M., Maneenil G., Dissaneevate S., Singkhamanan K., Surachat K. (2025). Characterization of Gut Microbiota in Very Low Birth Weight Infants with Versus without Bronchopulmonary Dysplasia. Clin. Exp. Pediatr..

